# Lower Health Literacy Is Associated with Poorer Diet Quality in Korean Adults: An Association Masked by Age

**DOI:** 10.3390/nu18183082

**Published:** 2026-09-20

**Authors:** Hyunjoo Joo, Jonghyuk Choi

**Affiliations:** 1 Department of Preventive Medicine, College of Medicine, Dankook University, Cheonan 31116, Republic of Korea; joojoossi@gmail.com; 2 Research Institute of Healthcare Bigdata, College of Medicine, Dankook University, Cheonan 31116, Republic of Korea

**Keywords:** health literacy, diet quality, Korean Healthy Eating Index, KHEI, confounding, age, fruit and vegetables, health disparities, KNHANES

## Abstract

**Background/Objectives**: Diet quality is a leading modifiable determinant of non-communicable disease, yet its upstream, person-level determinants are seldom studied. We examined whether health literacy is associated with overall diet quality in a nationally representative Korean sample. **Methods**: This cross-sectional study analyzed 5353 adults aged ≥19 years from the 2024 Korea National Health and Nutrition Examination Survey. Health literacy, measured with a validated 10-item instrument, was analyzed as score-based categories, continuously, and as the official binary indicator; diet quality was the Korean Healthy Eating Index (KHEI, 0–100) from a single 24 h recall. Survey-weighted linear and logistic regression was used, with component, subgroup, sensitivity, and multiple imputation analyses. **Results**: The crude difference between the lowest (≤28 points) and highest (≥33 points) categories was positive (β = +1.41); adjustment for age alone inverted it (β = −2.76), and the fully adjusted difference was β = −1.70 (95% CI, −2.66 to −0.75; −0.13 SD), with higher odds of lowest tertile diet quality (OR = 1.37; 95% CI, 1.09 to 1.72; *p* = 0.007). Each 1-SD increment in health literacy corresponded to β = +0.77 (95% CI, 0.42 to 1.12). Component differences were negative for 12 of 14, but none survived false discovery rate correction. The association was more evident in men (women vs. men, interaction β = −1.03 per 1-SD increment; 95% CI, −1.67 to −0.40). Multiple imputation (m = 20; n = 5678) gave consistent estimates. **Conclusions**: Lower health literacy was associated with modestly poorer diet quality, an association masked and reversed by age until adjustment. The cross-sectional design precludes causal inference, the single-day assessment may not represent habitual intake, and component and subgroup findings are exploratory.

## 1. Introduction

Suboptimal diet is among the leading modifiable contributors to the global burden of non-communicable disease, accounting for a large share of cardiometabolic and cancer mortality [1]. In the Republic of Korea, overall diet quality varies across sociodemographic groups and clinical status, and population diet quality has shifted appreciably over the past two decades [2,3], underscoring the value of identifying actionable determinants of healthful eating. Yet nutritional epidemiology has concentrated on intakes and their downstream health effects, with comparatively little attention to the upstream, person-level capacities that allow people to obtain, interpret, and act on dietary guidance.

Health literacy—the ability to access, understand, appraise, and apply health information to make health decisions—is increasingly recognized as a social determinant of health [4,5]. Limited health literacy is associated with poorer chronic disease management, lower use of preventive services, and wider health inequalities [6]. There is a plausible mechanistic pathway to diet: translating dietary guidance into daily food choices requires reading and comparing nutrition labels, making informed food purchases, planning meals, and possessing the skills and confidence to prepare healthful foods—capacities that fall within the access, understanding, appraisal, and application domains by which health literacy is defined [5]. Consistent with this pathway, higher health literacy has been linked to higher Healthy Eating Index scores and lower sugar-sweetened beverage intake [7], to closer adherence to the Mediterranean diet [8], and to better adherence with the Dietary Approaches to Stop Hypertension pattern [9]; health and nutrition literacy have also been associated with greater fruit and vegetable consumption across several settings [10,11], and a recent systematic review reported generally positive, if heterogeneous, associations of health and food literacy with dietary choices [12]. Importantly, and unlike fixed sociodemographic determinants of diet, health literacy is potentially modifiable through education, labeling, and clinical communication, which makes it an attractive intervention target [4,6].

Dietary behaviors may not depend equally on knowledge and planning. Obtaining and preparing adequate fruits and vegetables plausibly draws on knowing the recommendations, selecting and purchasing perishable produce, and organizing meals around them, and nutrition knowledge is a consistent—though weak—correlate of fruit and vegetable intake [13]. Whether the moderation components of a diet quality index depend less on those same capacities is far less clear, and the answer is likely to be context-specific. In Korea, in particular, sodium reaches the plate largely through home and restaurant cooking rather than through packaged foods: seasonings alone supply roughly 46–48% of dietary sodium, and kimchi, soups and stews, and noodle broths account for much of the remainder [14,15]. Label-reading skill, therefore, maps onto sodium moderation far less directly in Korea than in food environments dominated by pre-packaged products. Cost and retail availability—rather than knowledge—also emerge as the dominant barriers to fruit and vegetable intake in East and Southeast Asia [16]. Against this uncertain background, we nonetheless prespecified, as a tentative hypothesis to be tested rather than as an established mechanism, that any health literacy–diet association would be more apparent in the adequacy components of overall diet quality—particularly the fruit and vegetable components—than in the moderation or energy balance components. Food choices in any case reflect more than individual capability, including socioeconomic circumstances, food accessibility, cultural dietary patterns, taste preferences, and the wider food environment.

Health literacy has already been linked to dietary behavior in many settings. A recent systematic review of 49 studies reported 42 positive, 28 null, and two negative associations across 72 estimates, with the most consistent findings among studies using comprehensive health literacy instruments [17]. Our purpose is, therefore, not to establish that such a relationship exists but to characterize it with a combination of setting and measures that has not previously been available. Three considerations motivated this analysis. First, evidence linking a validated, nationally representative measure of health literacy to a validated index of overall diet quality is lacking in Korea; earlier work has more often relied on brief screeners or on single food groups [7,10,11]. Second, the specific dietary components that might underlie any association remain uncharacterized at the population level. Third, effect modification has rarely been examined, although the literacy dimensions that track diet quality have been reported to differ between men and women in a comparable East Asian population [18]. The KNHANES has administered a validated community health literacy instrument since the 2023 cycle [19,20,21], and the 2024 cycle allows that instrument to be linked to the official diet quality index.

Using the 2024 KNHANES, we (i) examined the association between health literacy and diet quality, operationalized with the official Korean Healthy Eating Index (KHEI) [22,23]; (ii) examined which KHEI components contributed to the association, testing our a priori fruit and vegetable hypothesis; (iii) assessed effect modification by sex, age, and income; and (iv) evaluated robustness to potential confounders and to missing covariate data. Because age is associated with health literacy and with diet quality in opposite directions in Korea, we deliberately present the progression from crude to fully adjusted estimates so that the confounding structure is visible rather than implicit.

## 2. Materials and Methods

### 2.1. Study Design and Population

**Figure 1:**
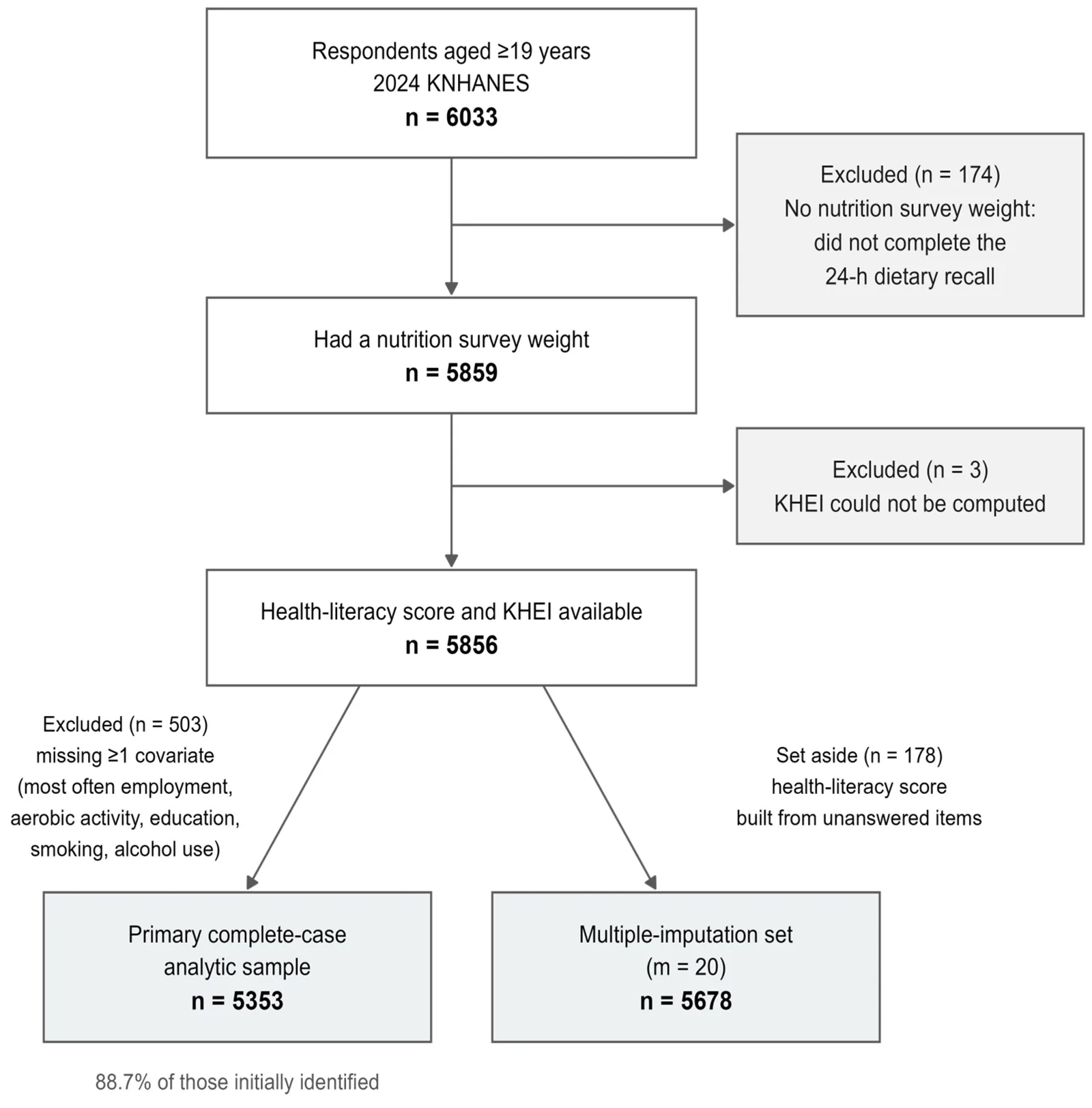
Flow of participants through the 2024 Korea National Health and Nutrition Examination Survey (KNHANES) into the two analytic samples. A nutrition survey weight was required because it is assigned only to respondents who completed the 24 h dietary recall from which the Korean Healthy Eating Index (KHEI) is derived. No participant was excluded for a missing health literacy score; the two respondents whose score was absent had already been excluded at the preceding step (Section 2.1). The 5856 respondents remaining after the KHEI step divide into the primary complete case analytic sample (n = 5353, after excluding 503 with a missing covariate) and the multiple imputation set (n = 5678, after setting aside 178 whose health literacy score was assembled from unanswered items, as explained in Section 2.2); the two samples overlap and are not mutually exclusive.

This study drew on the 2024 cycle of the Korea National Health and Nutrition Examination Survey (KNHANES), an annual survey run by the Korea Disease Control and Prevention Agency (KDCA) that samples the non-institutionalized Korean population through a stratified, multistage cluster design [24]. We started from 6033 respondents aged 19 years or older and then dropped those who lacked a nutrition survey weight because they had not completed a 24 h recall (n = 174), had no computable KHEI (n = 3), or were missing at least one covariate (n = 503; most commonly employment status, aerobic physical activity, educational attainment, smoking status, or alcohol use), leaving 5353 adults (88.7%) for the primary complete case analysis. The selection process is shown in Figure 1. All adults with a nutrition survey weight had a health literacy score; the two adults with a missing score had already been excluded at the preceding step. The 5856 respondents with a computable KHEI and a nutrition survey weight formed the pool for the missing data analyses described in Section 2.5, of whom 5678 also had a health literacy score reflecting answered items. The KNHANES protocol was approved by the KDCA Institutional Review Board, and every respondent gave written informed consent. Because we worked only with de-identified public use files, this secondary analysis did not call for separate ethics review.

### 2.2. Health Literacy (Exposure)

We measured health literacy with the 10-item Health Literacy Index for the Community, a standardized tool developed for the KNHANES and administered since the 2023 cycle, which has been validated for use in this population [19,20]. Its items ask how readily a respondent can obtain health information, make sense of it, judge whether it can be trusted, and act on it when making health decisions. Every item is answered on a 4-point scale, and the responses add up to a single score that runs from 10 to 40, with higher totals marking stronger health literacy. One feature of the summary score distributed with the survey should be noted: each item carries a code for “don’t know or no response”, and that code enters the summary score as a value of one. A respondent who answered none of the items, therefore, receives the minimum possible score of 10 rather than a missing value, and a respondent who left some items unanswered receives an intermediate score that understates measured literacy. Across the 6033 respondents initially identified, 246 have a score constructed in this way, and a further two have no score at all, so that 248 (4.1%) lack a measured score. They are heavily concentrated among respondents who were excluded for other reasons, and only 14 of the 5353 respondents in the analytic sample (0.26%) are affected; the consequences are considered in Section 3.3 and Section 4.6. Because the score is discrete and many participants share identical values—most notably 30 points, held by 28% of respondents—rank-based quartiles would place participants with the same score in different categories. We, therefore, defined four score-based categories that keep tied scores together, using the cut-points of the observed quartile distribution (28, 30, and 32 points): ≤28 (n = 1465; 25.5% weighted), 29–30 (n = 2018; 37.0%), 31–32 (n = 480; 9.3%), and ≥33 (n = 1390; 28.1%). Category membership is, therefore, fully determined by the score, at the cost of unequal category sizes, which the large modal value makes unavoidable. The highest category (≥33 points) served as the reference. These four categories are defined by the observed distribution of scores in this sample; they are not clinically validated strata, and the boundaries would not necessarily transfer to another population. For that reason, we also analyzed health literacy continuously—per point and per standard deviation (1 SD = 4.99 points in the analytic sample)—and as the officially defined binary indicator (≥30 of 40 points = adequate health literacy), the national indicator used in Korea’s Health Plan 2030. Both specifications are reported alongside the four-category analysis rather than being confined to a sensitivity analysis. Further sensitivity analyses used alternative tie-respecting category definitions (≤26/27–29/30–31/≥32 points, and <30/=30/>30 points, each with the highest category as the reference); these retained all participants, and the lowest category was compared with the highest under each definition.

### 2.3. Diet Quality (Outcome)

Diet quality was captured with the Korean Healthy Eating Index (KHEI), an index built for KNHANES to grade how closely a person’s intake tracks the national dietary guidelines [22,23]. It rates 14 items on a 0–100 scale and sorts them into three domains. Eight items cover adequacy: eating breakfast, mixed grains, total fruit, fresh fruit, total vegetables, vegetables excluding kimchi, the meat–fish–egg–bean group, and milk or dairy. Three cover moderation: the share of energy from saturated fat, sodium, and the share of energy from sweets and beverages. The last three cover balance: the share of energy from carbohydrates and from fat, together with whether total energy intake falls in an appropriate range. Instead of re-scoring the recall ourselves, we took the total and component KHEI values that the KDCA derives from the 24 h recall and distributes with the dataset. Because only one recall day was available per respondent, the KHEI used here represents diet quality estimated from a single day of reported intake rather than an assessment of habitual diet; day-to-day within-person variation is, therefore, embedded in the outcome [25]. As a secondary and more easily read outcome, we defined lowest tertile diet quality as falling in the lowest survey-weighted tertile of the KHEI (total score ≤ 52.9), while keeping the continuous score as the primary outcome. This cut-off describes the lowest third of the weighted KHEI distribution in the present study population; it is not an externally validated or clinically meaningful threshold, and the corresponding logistic analysis is reported as a secondary check on the robustness of the continuous results rather than as a clinical classification.

### 2.4. Covariates

**Figure A1:**
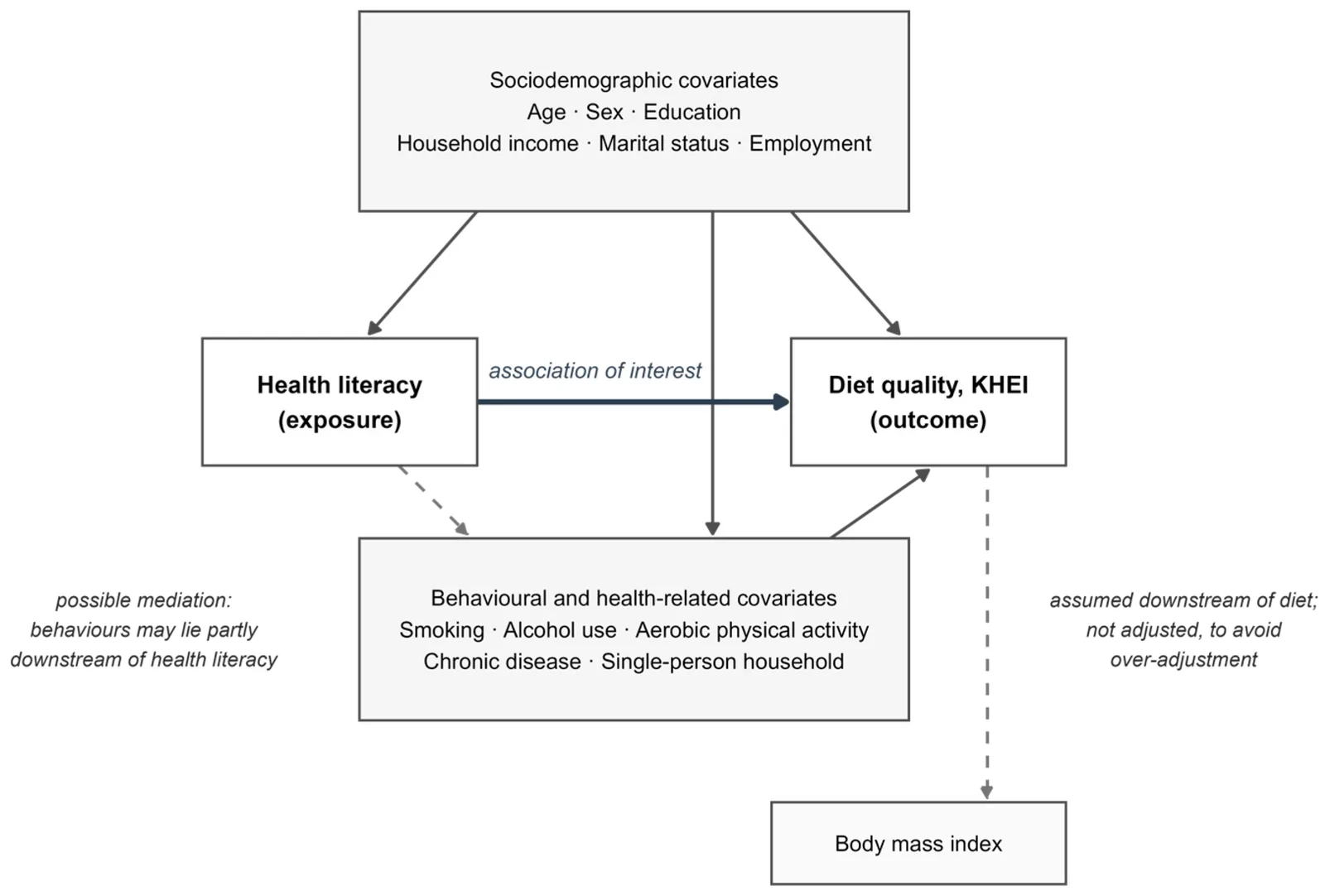
Directed acyclic graph (DAG) showing the relationships assumed among health literacy, diet quality (KHEI), and the covariates. Solid arrows mark relationships assumed from previous literature and subject matter reasoning; dashed arrows mark assumed relationships that motivated an analytic choice rather than an adjustment. Sociodemographic covariates are treated as common causes of health literacy and diet quality and form the Model 2 adjustment set. The behavioral and health-related covariates are added in Model 3 but are drawn as potentially downstream of health literacy, which is why Model 3 is interpreted as an association net of those pathways rather than as a total effect. Body mass index is assumed to lie downstream of diet and was, therefore, not adjusted for, to avoid over-adjustment. The arrows encode assumptions, not findings: health literacy, diet, and every covariate were measured at the same cross-sectional examination, so none of these directions can be established from the present data.

Covariates were selected a priori using a directed acyclic graph (DAG; Figure A1) and comprised sex; age; educational attainment; household income quintile; marital status; employment status; smoking status (current versus non-current, that is, former or never smoker); lifetime alcohol use (ever consumed ≥1 alcoholic drink; KNHANES item bd1); aerobic physical activity; physician-diagnosed chronic disease (defined as physician-diagnosed hypertension and/or diabetes mellitus, the most prevalent diet-sensitive conditions captured by KNHANES); and single-person household status. Body mass index (BMI) was deliberately not included: it plausibly lies downstream of diet (diet → adiposity), so conditioning on it would constitute over-adjustment. Depressive symptoms (Patient Health Questionnaire-9 [PHQ-9] score ≥ 10) [26], perceived stress, and total energy intake were reserved for sensitivity analyses.

### 2.5. Statistical Analysis

All estimates were produced under the survey’s complex design, applying the integrated nutrition survey weights along with the stratum and primary sampling unit codes, as KNHANES recommends for recall-based dietary data. We first summarized participants by health literacy category, reporting survey-weighted means and standard deviations for continuous variables and unweighted counts with survey-weighted percentages for categorical variables. The KHEI was then regressed on health literacy in three survey-weighted linear models fitted in sequence: an unadjusted model (Model 1); a model that added sex, age, education, income, marital status, and employment (Model 2); and a model that further added smoking, alcohol use, aerobic physical activity, chronic disease, and single-person household status (Model 3). To isolate the contribution of age, we additionally fitted a model adjusted for age alone (Model 1a). Health literacy was also entered continuously, per point and per standard deviation, to look for a linear trend, and the odds of lowest tertile diet quality were estimated with survey-weighted logistic regression. To place the size of the association on an interpretable scale, the fully adjusted difference between the lowest and highest categories was additionally expressed in standard deviation units of the survey-weighted KHEI distribution (weighted SD = 13.06) [27]. Each of the 14 KHEI components was treated as its own fully adjusted outcome; because the maximum attainable score differs across components (5 or 10 points), component-level differences are additionally expressed as a percentage of each component’s maximum score so that components can be compared on a common scale. For the 14 component-level tests, we calculated Benjamini–Hochberg false discovery rate-adjusted *p* values [28]; subgroup analyses were not multiplicity-corrected. Effect modification by sex, age band, and income was tested with stratified models and multiplicative interaction terms assessed by Wald tests. For the continuous specification, the interaction estimate is reported with its 95% confidence interval; for the categorical exposure, which contributes three interaction terms, the joint Wald *p* value is reported. Age bands were defined as 19–39, 40–64, and ≥65 years to represent younger, middle-aged, and older adults and to align with the working age and retirement transitions relevant to Korean dietary patterns; age was entered as a continuous variable in all multivariable models, and these bands were used only for description and for exploratory subgroup analysis. Component and subgroup analyses were prespecified but regarded as exploratory. Further models adjusted for depressive symptoms and perceived stress, adjusted for total energy intake, and repeated the primary analysis after excluding implausible energy reports below 500 or above 5000 kcal/day. Model 2 provided the sociodemographic-adjusted estimate, whereas Model 3 additionally adjusted for behavioral and health-related characteristics to assess the robustness of the association to broader covariate control. Because some of these additional characteristics may partly lie downstream of health literacy, estimates from both models were interpreted together, and Model 3 is reported as the main adjusted estimate.

Because complete case analysis excluded 680 of the 6033 respondents initially identified (11.3%), two further analyses addressed missing data. First, we compared included and excluded respondents on health literacy score, age, and—where the KHEI could be computed—crude diet quality, and calculated the exclusion rate within each health literacy category. Second, we performed a multiple imputation sensitivity analysis. Because a score assembled from unanswered items understates measured literacy and would introduce exposure misclassification (Section 2.2), the imputation set comprised the 5678 respondents who had a nutrition survey weight, a computable KHEI, and a health literacy score reflecting answered items; within that set, 317 were missing employment status, 316 aerobic physical activity, 16 household income, and 3 educational attainment. Twenty imputed datasets were generated by chained equations (polytomous and binary logistic models for categorical covariates), with the exposure, the outcome, all analysis covariates, and the survey design information included in the imputation model so that the imputation and analysis models remained congenial [29,30]. Each imputed dataset was analyzed under the full KNHANES complex survey specification, and estimates were combined using Rubin’s rules. As a further check, the procedure was repeated across all 5856 respondents with a nutrition survey weight and a computable KHEI, imputing the health literacy score itself for the 178 whose score did not reflect answered items. All analyses were performed in R version 4.5.2 (R Foundation for Statistical Computing, Vienna, Austria) using the survey package (version 4.5) and the mice package (version 3.19.0); a two-sided *p* < 0.05 was treated as significant.

## 3. Results

### 3.1. Participant Characteristics

The analytic sample comprised 5353 adults; the survey-weighted mean KHEI was approximately 59 points (weighted tertile cut points, 52.9 and 64.7). The survey-weighted prevalence of each health literacy category was 25.5% for scores ≤ 28, 37.0% for 29–30, 9.3% for 31–32, and 28.1% for ≥33, corresponding to unweighted counts of 1465, 2018, 480, and 1390 respondents. Participants in the lowest health literacy category (≤28 points) were markedly older (mean 55.0 vs. 43.5 years) and less educated (28.6% vs. 62.3% college educated) than those in the highest category (≥33 points), and they were more often physically inactive, living alone, and living with chronic disease (Table 1). Across age groups, survey-weighted mean KHEI scores increased from 51.7 among adults aged 19–39 years to 59.6 among those aged 40–64 years and 67.0 among those aged ≥65 years, whereas mean health literacy scores decreased from 31.9 to 31.0 and 28.5, respectively. Tellingly, crude KHEI differed across the four categories (global *p* = 0.001), with a slightly higher mean in the lowest than in the highest category (59.2 vs. 57.8)—the raw manifestation of the negative confounding by age examined in the next section.

### 3.2. Health Literacy and Diet Quality

**Figure 2:**
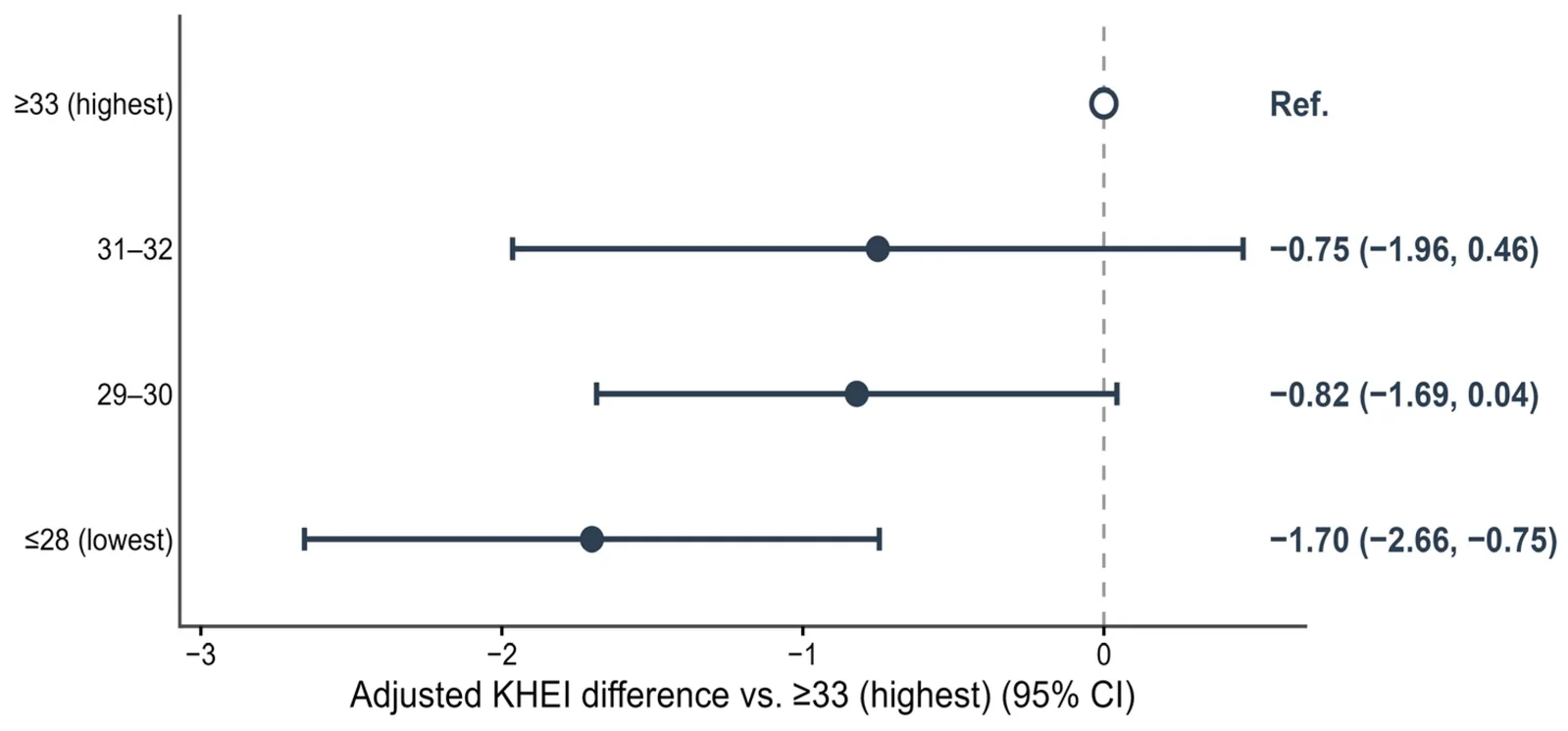
Association between health literacy category and diet quality (KHEI), fully adjusted (Model 3). Points are adjusted mean differences versus the highest category (≥33 points), with horizontal bars showing 95% confidence intervals; the reference category is drawn as an open marker. Numerical estimates and confidence intervals are printed beside each row.

**Figure 3:**
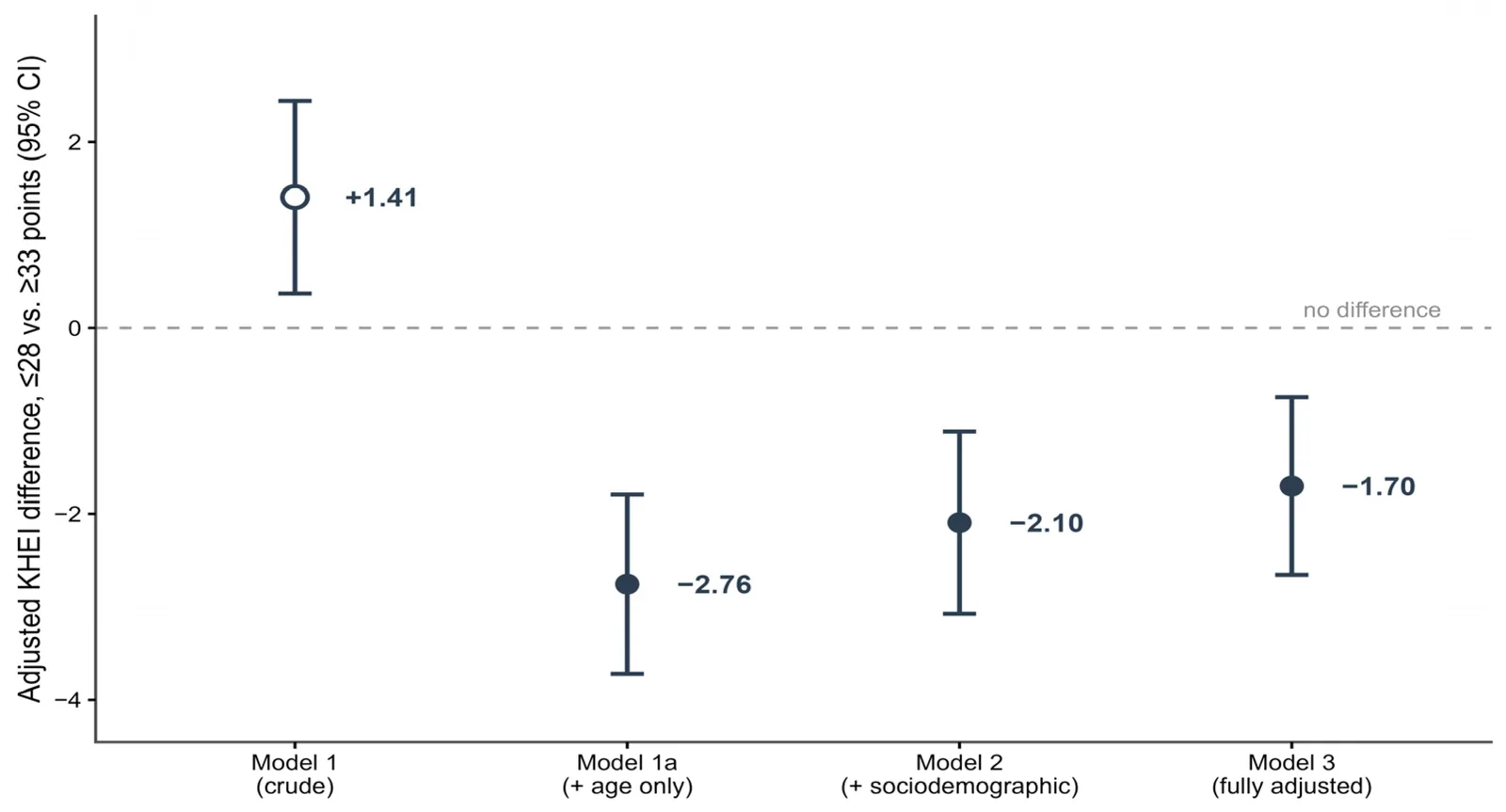
Estimated health literacy–diet quality difference under successive covariate adjustment specifications. Estimates are adjusted mean differences in KHEI for the lowest (≤28 points) versus the highest (≥33 points) health literacy category, with 95% confidence intervals. The crude estimate (Model 1, drawn as an open marker) is positive; adjustment for age alone (Model 1a) already inverts its direction, and further adjustment for other sociodemographic factors (Model 2) and for health behavior and chronic disease (Model 3) partially attenuates it. All four values are observational regression estimates obtained under different covariate sets; the sequence illustrates how sensitive the estimate is to adjustment for age and is not a progression towards a causal effect.

In the unadjusted model, lower health literacy appeared paradoxically associated with higher KHEI (lowest vs. highest category, β = +1.41; 95% CI, 0.37 to 2.44). This crude estimate inverted after adjustment for age and sociodemographic factors (Model 2: β = −2.10; 95% CI, −3.07 to −1.12) and persisted after full adjustment (Model 3: β = −1.70; 95% CI, −2.66 to −0.75; Figure 2, Table 2). Expressed on a standardized scale, that fully adjusted difference corresponds to −0.13 SD (95% CI, −0.20 to −0.06) of the survey-weighted KHEI distribution. Fully adjusted differences were more negative in the lower categories and most markedly so in the lowest (31–32 points, β = −0.75; 29–30 points, β = −0.82; ≤28 points, β = −1.70); the two intermediate estimates were close to one another, and only the lowest category differed significantly from the highest. Adjustment for age alone was sufficient to reverse the crude estimate (Model 1a: β = −2.76; 95% CI, −3.72 to −1.79; *p* < 0.001), yielding a difference larger in magnitude than either subsequent model. Because older adults had both lower health literacy and higher diet quality, age operated as a strong negative confounder that masked and reversed the observed association until it was controlled; the estimate attenuated further as sociodemographic and behavioral characteristics were added in Models 2 and 3 (Figure 3). Analyzed continuously—the specification that does not depend on distribution-based category boundaries—health literacy showed a positive association with the KHEI on the continuous scale (β = +0.15 per 1 point; 95% CI, 0.08 to 0.22; β = +0.77 per 1 SD; 95% CI, 0.42 to 1.12; both *p* < 0.001). Adults in the lowest category also had higher odds of lowest tertile diet quality than those in the highest (OR = 1.37; 95% CI, 1.09 to 1.72; *p* = 0.007).

### 3.3. Missing Data and Multiple Imputation

Of the 6033 respondents initially identified, 680 (11.3%) were excluded. Because a score built from unanswered items understates measured literacy rather than marking it, the comparison of included and excluded respondents is restricted to those whose score reflects answered items: 14 respondents in the analytic sample and 234 among those excluded are set aside for this purpose (Table A3). On that basis, respondents excluded for missing covariate data had modestly lower mean health literacy scores than those included (29.1 vs. 30.4 points) and were somewhat older (56.6 vs. 53.5 years), while their crude KHEI was very similar (60.7 vs. 60.2 points); respondents excluded for lacking a nutrition survey weight were comparable (29.4 points; 55.3 years). Within-category exclusion rates were 9.8% among adults scoring ≤28 points, against 7.8%, 5.0%, and 6.3% in the 29–30, 31–32, and ≥33 categories (Table A4). Exclusion was, therefore, related to the exposure, but mildly rather than steeply. Had the scores built from unanswered items been read at face value, the same comparison would have suggested a far larger deficit among excluded respondents (23.1 vs. 30.4 points) and a far steeper exclusion gradient (21.0% in the lowest category), neither of which reflects measured health literacy.

In the multiple imputation analysis (m = 20; n = 5678), estimates were close to the complete case results throughout (Table A5). The adjusted KHEI difference between the lowest and highest categories was −1.66 points (95% CI, −2.57 to −0.74), against −1.70 (95% CI, −2.66 to −0.75) in the complete case analysis. Each 1 SD increment in health literacy corresponded to +0.76 KHEI points (95% CI, 0.44 to 1.08) versus +0.77 (95% CI, 0.42 to 1.12); limited health literacy under the official binary threshold corresponded to −0.97 points (95% CI, −1.67 to −0.27) versus −1.06 (95% CI, −1.80 to −0.32); and the odds ratio for lowest tertile diet quality was 1.33 (95% CI, 1.07 to 1.64) versus 1.37 (95% CI, 1.09 to 1.72). Two estimates crossed the conventional significance threshold in the process: the difference for the 29–30 category, which was −0.97 (95% CI, −1.80 to −0.13) after imputation against −0.82 (95% CI, −1.69 to 0.04) in the complete case analysis, and the odds ratio for the official binary indicator, 1.15 (95% CI, 0.98 to 1.35) against 1.20 (95% CI, 1.01 to 1.42). Imputing the health literacy score as well, across all 5856 respondents with a nutrition survey weight and a computable KHEI, gave closely similar results (lowest versus highest category, −1.67; 95% CI, −2.58 to −0.75). Restoring the respondents excluded for incomplete covariate data, therefore, neither strengthened nor materially weakened the association.

### 3.4. Component-Level Associations

**Figure 4:**
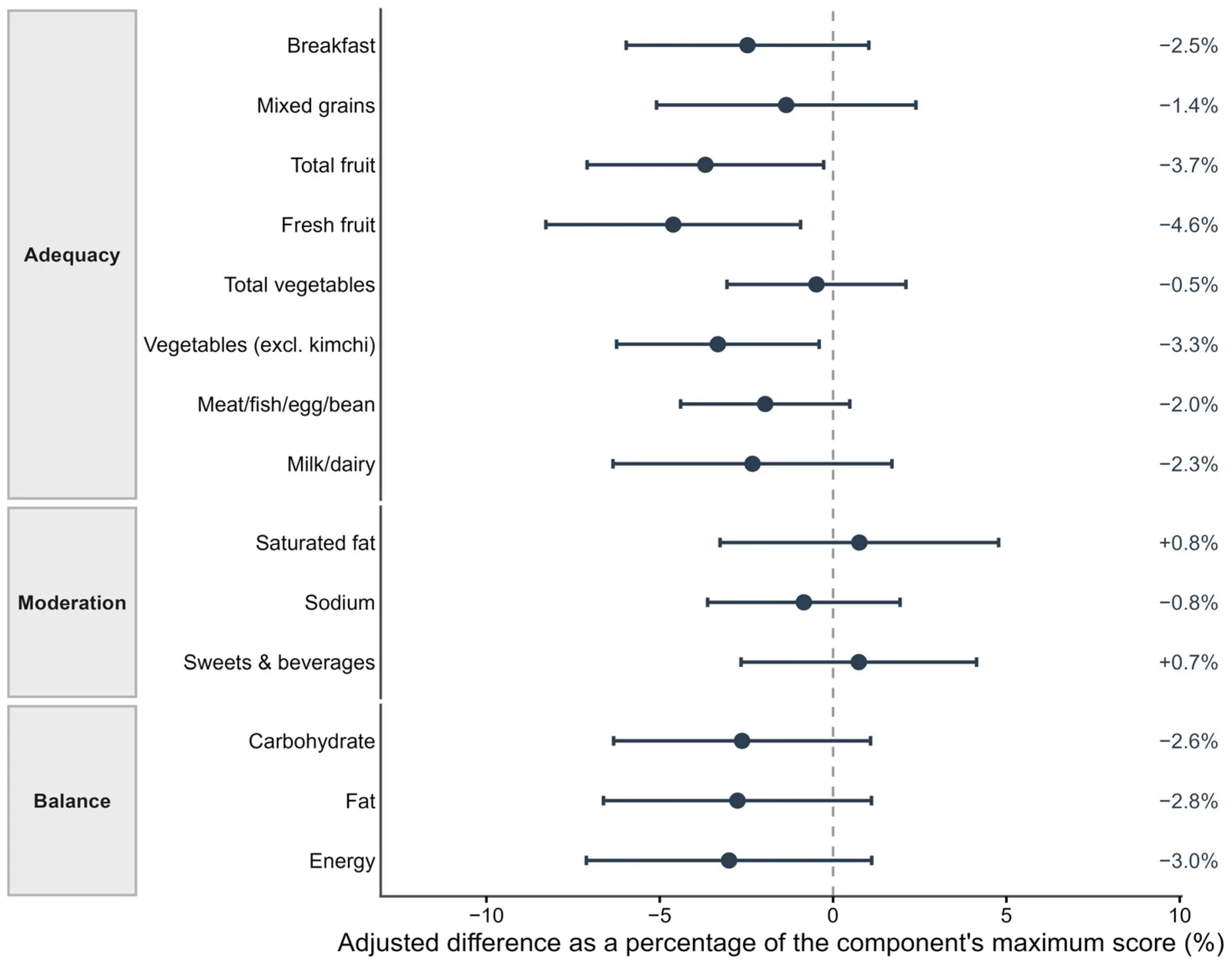
Adjusted differences in each KHEI component score for the lowest versus highest health literacy category (≤28 vs. ≥33 points), expressed as a percentage of each component’s maximum score because the maximum attainable score differs across components (5 or 10 points). Horizontal bars are 95% confidence intervals; shaded bands group components by domain (adequacy, moderation, balance). No component remained statistically significant after false discovery rate correction, and the figure, therefore, does not mark nominal significance. The corresponding figure in raw component score units is provided as Figure A2.

**Figure A2:**
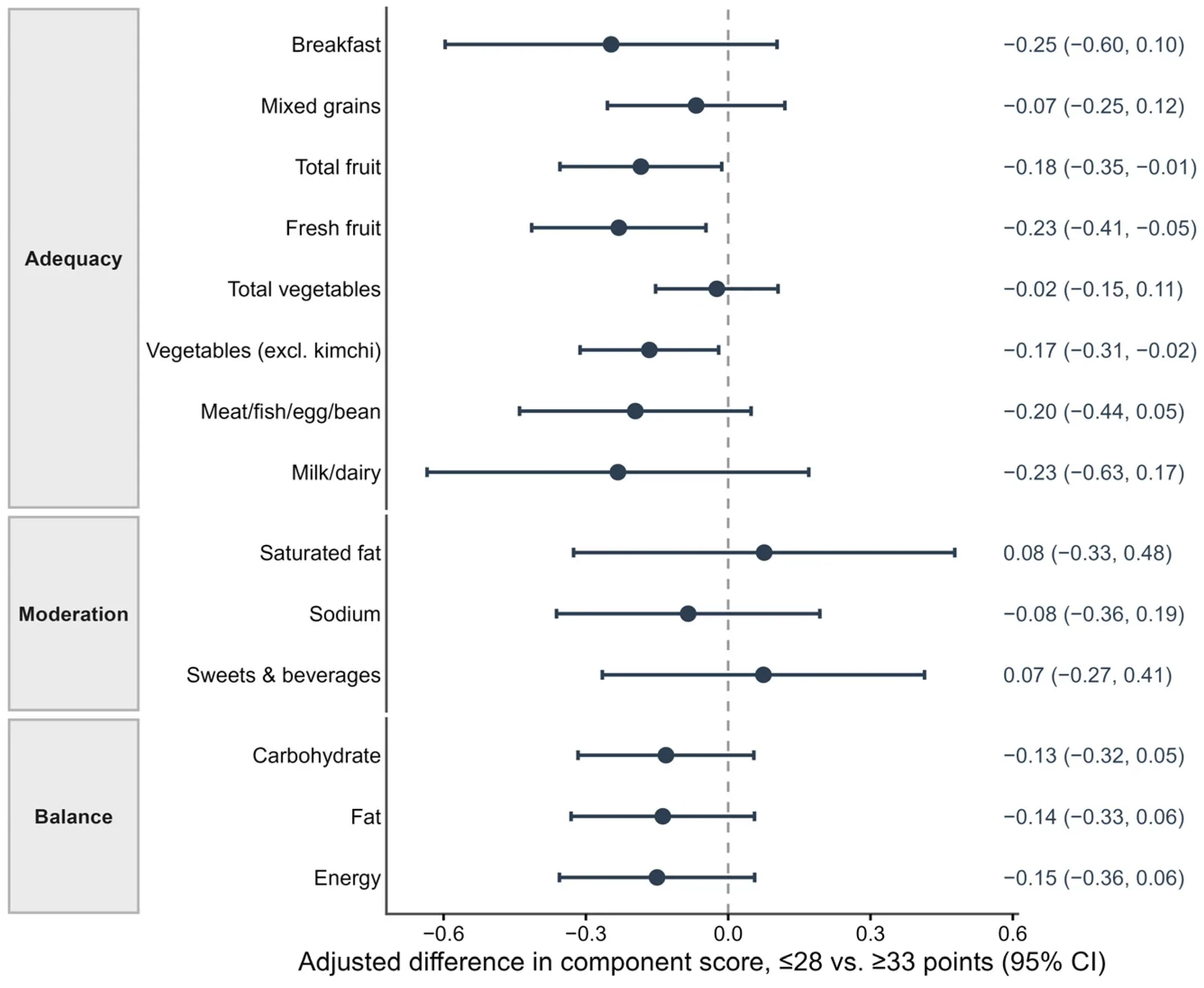
Adjusted differences in each KHEI component score for the lowest versus highest health literacy category (≤28 vs. ≥33 points), expressed in raw component score units. Horizontal bars are 95% confidence intervals; shaded bands group components by domain (adequacy, moderation, balance). As in Figure 4, the figure does not mark nominal significance because no component remained significant after false discovery rate correction. Because the maximum attainable score differs across components (5 or 10 points), raw differences are not directly comparable across components; the corresponding figure scaled to each component’s maximum is Figure 4.

The component-level results are shown in Figure 4 and Table A1; the same estimates in raw component score units are shown in Figure A2. The largest relative deficits in the lowest health literacy category were in fruit and vegetable adequacy—fresh fruit (β = −0.23; 95% CI, −0.41 to −0.05), total fruit (β = −0.18; 95% CI, −0.35 to −0.01), and vegetables excluding kimchi (β = −0.17; 95% CI, −0.31 to −0.02), equivalent to −4.6%, −3.7%, and −3.3% of each component’s maximum score—and these were the only components with an unadjusted *p* < 0.05. After Benjamini–Hochberg correction across the 14 component tests, however, no component remained significant (all false discovery rate-adjusted *p* ≥ 0.16). Differences were negative for 12 of the 14 components; the three fruit and vegetable components accounted for −0.58 points (34% of the total −1.70 point difference), although they carry only 15 of the 100 possible KHEI points. No component survived correction for multiple testing, however, so the component-level pattern is reported as exploratory rather than as evidence that the deficit is localized. Because no component survived multiplicity correction, the a priori fruit and vegetable hypothesis was not confirmed, and these component-level findings are hypothesis-generating only.

### 3.5. Subgroup Analyses

**Figure 5:**
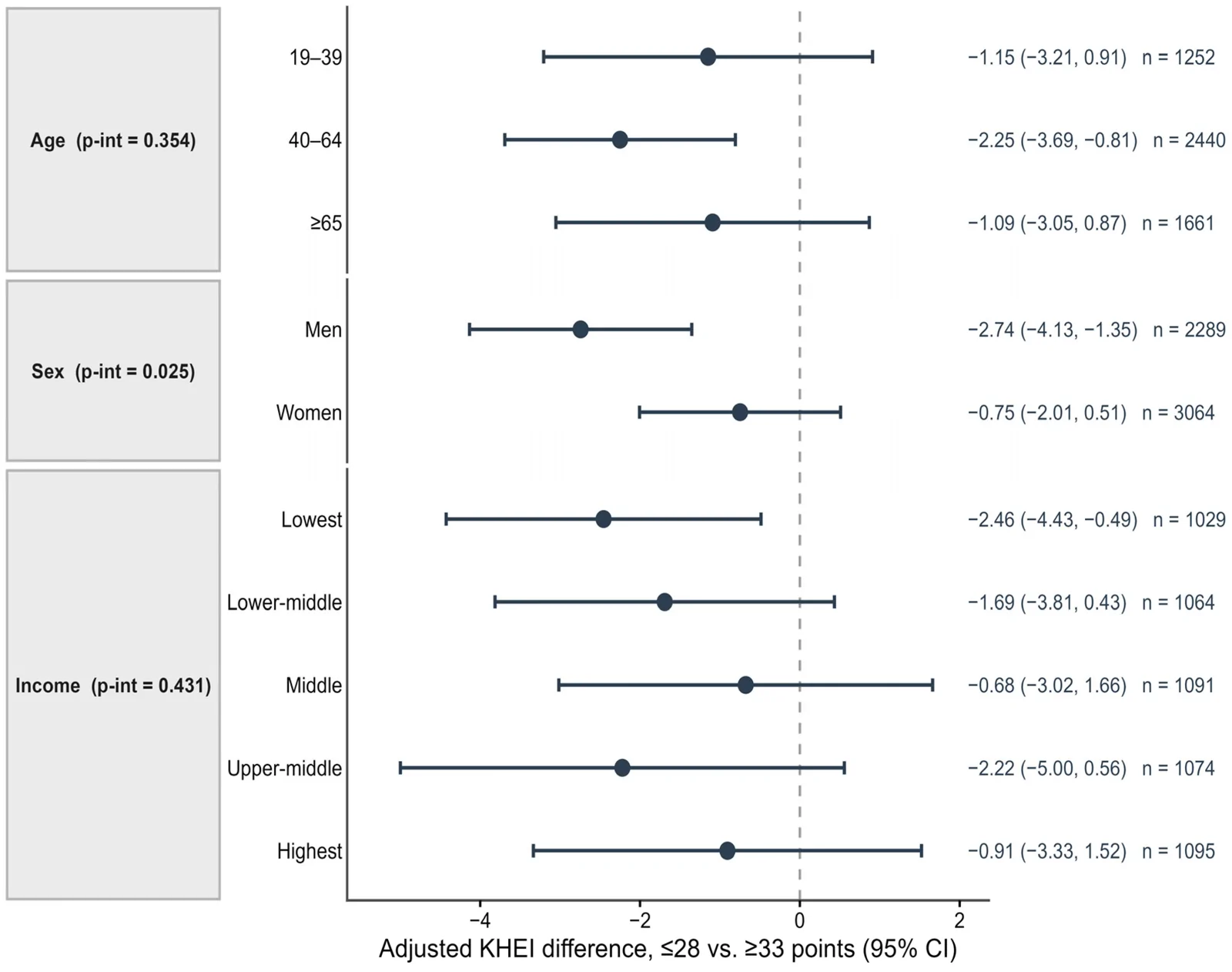
Adjusted difference in KHEI for the lowest versus highest health literacy category within subgroups defined by age, sex, and income, with 95% confidence intervals, subgroup sample sizes, and joint Wald interaction *p* values. Estimates come from the fully adjusted model fitted within each stratum. Subgroup analyses were not corrected for multiple comparisons and are exploratory.

The association was more evident in men (β = −2.74; 95% CI, −4.13 to −1.35) than in women (β = −0.75; 95% CI, −2.01 to 0.51), and the multiplicative interaction across the four categories was statistically significant (joint Wald *p* = 0.025; Figure 5). In the continuous specification, the interaction coefficient for women relative to men was −1.03 KHEI points per 1-SD increase in health literacy (95% CI, −1.67 to −0.40; *p* = 0.002). By age, the association was strongest at 40–64 years (β = −2.25; 95% CI, −3.69 to −0.81), although the interaction was not significant (*p* = 0.354). By income, a significant association appeared only in the lowest group (β = −2.46; 95% CI, −4.43 to −0.49), again without significant interaction (*p* = 0.431). Because several potential modifiers were examined without correction for multiplicity, all subgroup results, including the sex difference, are treated as exploratory (Table A2).

### 3.6. Sensitivity Analyses

The primary association was stable across sensitivity analyses. Additional adjustment for depressive symptoms and perceived stress, in the 5208 participants with a complete PHQ-9 (β = −1.50; 95% CI, −2.49 to −0.52), for total energy intake (β = −1.74; 95% CI, −2.69 to −0.79), and restriction to plausible energy reporters (β = −1.63; 95% CI, −2.59 to −0.67) left estimates essentially unchanged. Using the officially defined binary indicator, adults with limited health literacy (<30 points) had a lower KHEI than those with adequate literacy (β = −1.06; 95% CI, −1.80 to −0.32) and higher odds of lowest tertile diet quality (OR = 1.20; 95% CI, 1.01 to 1.42). Estimates were directionally consistent in alternative models that retained all intermediate score categories (≤26 vs. ≥32 points, β = −2.41; 95% CI, −3.54 to −1.27; <30 vs. >30 points, β = −1.32; 95% CI, −2.15 to −0.50), although effect magnitudes varied across category definitions. Restricting the analysis to the 5339 respondents whose health literacy score reflects answered items (Section 2.2) also left the primary estimate essentially unchanged (β = −1.69; 95% CI, −2.64 to −0.73).

## 4. Discussion

### 4.1. Principal Findings

In this nationally representative sample of Korean adults, lower health literacy was associated with modestly poorer overall diet quality after extensive covariate adjustment. The association was positive on the continuous literacy scale, largest in the lowest score category, reproduced by the officially defined binary indicator, and reflected in higher odds of lowest tertile diet quality. Three features define the result: it was masked and inverted by age until adjustment, it was more evident in men, and it could not be attributed to any single dietary component. That health literacy and diet are related is already well documented [12,17]; what this analysis adds is set out in Section 5.

### 4.2. Age as a Negative Confounder

The crude analysis was not merely attenuated but reversed in sign. Adults in the lowest health literacy category appeared to have better diet quality than those in the highest (β = +1.41; 95% CI, 0.37 to 2.44), yet adjustment for age alone moved the estimate to β = −2.76 (95% CI, −3.72 to −1.79)—a swing of more than four KHEI points from a single covariate. Further adjustment for other sociodemographic characteristics (Model 2, β = −2.10) and for health behaviors and chronic disease (Model 3, β = −1.70) attenuated the estimate but did not alter its direction (Figure 3). Participants in the lowest category were on average 11.5 years older than those in the highest (55.0 vs. 43.5 years). Consistent with previous Korean studies, health literacy was lower at older ages [20,21,31,32], whereas age was positively associated with the KHEI in our data. When a covariate is associated with the exposure and the outcome in opposite directions, the observed association can shrink, vanish, or reverse on adjustment—the reversal paradox [33]—and the age gradients here were strong enough to produce a full reversal. The same configuration has been reported outside Korea: in a Slovenian national survey, older age predicted lower health literacy but higher fruit and vegetable intake [34], and a meta-analysis of aging and functional health literacy documents a steep decline in comprehension-based literacy with age [35].

The opposing age patterns themselves have precedent in Korean data. Age shows the reverse pattern for diet quality: a previous age–period–cohort analysis of KNHANES 2007–2018 found that Korean Healthy Eating Index scores were lowest in the late thirties and rose steadily with age up to 79 years, independently of survey period and birth cohort, and that diet quality peaked in the cohort born in the early 1960s and declined in each later cohort, consistent with earlier lifetime exposure to a Westernized food environment [3]. A more recent age–period–cohort analysis of Korean potassium intake likewise found age and cohort effects running in opposite directions [36]. Because age in a cross-sectional survey indexes birth cohort, education, socioeconomic circumstances, dietary culture, and health behavior simultaneously, the present analysis cannot separate an age effect from a generational one, and we do not claim that the age-adjusted estimate recovers a uniquely correct value.

Two practical implications follow. First, a single fully adjusted estimate can conceal how much of a result depends on one covariate; reporting the full crude-to-adjusted progression, as we do in Table 2 and Figure 3, makes the confounding structure visible and auditable. Had we reported only the crude association, we would have concluded—incorrectly—that lower health literacy accompanies better diet quality. Second, the progression is itself informative: the attenuation from Model 1a to Model 2 indicates that conventional indicators of socioeconomic position account for part of the age-adjusted association, consistent with confounding or shared variation by education and income; a comparable attenuation by educational attainment has been reported for health literacy and DASH adherence in a US sample [9].

### 4.3. Component-Level Findings

We had hypothesized a priori that any association would be concentrated in the fruit and vegetable components. The largest relative deficits were indeed observed for fresh fruit (−4.6%), total fruit (−3.7%), and vegetables excluding kimchi (−3.3%), and these were the only components with an unadjusted *p* < 0.05; however, none survived correction for multiple testing, and differences were negative for 12 of the 14 components. The hypothesis is, therefore, not confirmed, and the component-specific pattern remains exploratory rather than established. Two considerations caution against reading component specificity into these data. First, achieving adequate fruit and vegetable intake does plausibly draw on knowledge, planning, and preparation skills, but nutrition knowledge is only a weak correlate of intake even where it is measured directly [13], and in East and Southeast Asian settings, cost and availability dominate the reported barriers [16]. Second, the assumption that moderation behaviors are governed mainly by an obesogenic packaged food environment does not describe Korea well: sodium here arises overwhelmingly from seasonings, kimchi, soups and stews, and noodle broths prepared at home or in restaurants rather than from pre-packaged products [14,15], so label-reading skill would not be expected to translate into better sodium scores in the first place. The null component-level findings are thus compatible with several accounts, and we do not treat them as evidence that health literacy shapes what people add to their diets more than what they avoid.

### 4.4. Sex Differences

Few population-based studies have evaluated sex differences in the health literacy–diet relationship, and the more pronounced male pattern we observed is, to our reading, not well established. Higher diet quality among women than among men has been reported in other populations [37], and a 23-country study found that sex differences in food choice were substantially explained by health beliefs and dieting rather than by sex itself [38]. In a nationwide Japanese sample, the food literacy dimensions associated with overall diet quality differed between men and women, with nutrition knowledge and cooking skills associated with diet quality in women only [18]—a pattern that does not simply mirror ours and underlines how unsettled this question remains. Korean data also indicate that the correlates of health literacy differ by sex [39]. One speculative explanation for our finding is that where dietary decisions depend more on self-directed acquisition and appraisal of nutrition information, limited health literacy may translate more directly into poorer diet quality; we did not measure information-seeking behavior and advance this only as a hypothesis. Given that several modifiers were tested without multiplicity correction, we regard the sex difference as a potential effect modification requiring confirmation in independent and, ideally, longitudinal samples rather than as an established male-specific association.

### 4.5. Public Health Significance

Whatever its distribution across subgroups, the association is small. The difference between the lowest and highest health literacy categories was, in absolute terms, about 1.7 points on a 100-point scale, or −0.13 SD (95% CI, −0.20 to −0.06). By conventional benchmarks, this is a small effect, and at the individual level it is unlikely to be perceptible. Its population interpretation may nevertheless differ, because small standardized differences that apply to a large share of the population can accumulate into non-trivial aggregate consequences [27], and under Rose’s prevention paradox [40], a modest downward shift distributed across a large, low-literacy segment of the population can generate more aggregate disease than large deficits in a few individuals. This remains an argument about plausibility rather than a demonstration: the present study measured neither disease outcomes nor the effect of any intervention, and we cannot show that raising health literacy would raise KHEI scores.

Health literacy is nevertheless a modifiable characteristic, which distinguishes it from fixed sociodemographic markers of dietary disadvantage, and it is addressable through familiar, non-coercive channels, such as nutrition labeling and community- and workplace-based nutrition education, and brief dietary counselling in primary care. The evidence that such measures change what people eat is, however, mixed: a meta-analysis of front-of-pack labeling found modest improvements in the nutrient composition of purchases but limited and inconsistent effects on measured consumption [41], and fruit and vegetable intake in this region—whose health benefits are well documented [42]—appears to be constrained at least as much by cost and availability as by information [16]. These approaches are, therefore, best described as candidates for evaluation rather than as implications of the present findings, which are cross-sectional and cannot establish that any of them would improve diet quality.

### 4.6. Strengths and Limitations

Strengths include the nationally representative design with appropriate survey weighting, use of the official KHEI (avoiding investigator scoring), a newly validated health literacy instrument, component-level resolution with multiplicity control, formal tests of effect modification, and a multiple imputation analysis addressing differential exclusion. Several limitations apply. First, the cross-sectional design precludes causal and temporal inference: health literacy and diet quality were measured at the same moment, and the data cannot show that one preceded the other. Reverse pathways are plausible—people with established health conditions or greater health engagement may both seek out nutrition information and eat more healthfully, and better diet may itself foster further information seeking—as are shared determinants: nutritional vulnerability and depressive symptoms frequently co-occur among Korean adults, including those living alone [43], which motivated our adjustment for depressive symptoms and perceived stress in the sensitivity analyses. Second, diet was assessed with a single 24 h recall, which captures one day’s intake rather than usual intake. Within-person day-to-day variability and reporting error are, therefore, embedded in the outcome [25]. For the continuous KHEI, this adds noise and widens the confidence interval rather than biasing the coefficient, but for the tertile-based secondary outcome, it blurs the classification and would be expected to move the odds ratio towards the null. Third, health literacy and most covariates were self-reported. Because both the exposure and the dietary components were partly self-reported, shared method and social desirability biases could have inflated the association; in addition, the 10-item instrument, though validated, is brief and may incompletely capture the health literacy construct.

Missing data deserve particular emphasis. Complete case analysis excluded 680 of 6033 respondents (11.3%), and the exclusion was related to the exposure: among respondents whose health literacy score reflects answered items, 9.8% of those scoring ≤ 28 points were excluded, against 5–8% in each higher category, and excluded respondents were older and scored modestly lower on health literacy than those retained. Selection of this kind can bias an association in either direction, and its direction cannot be determined from the data alone without knowing how exclusion relates jointly to health literacy and diet quality. Multiple imputation gave estimates close to the complete case results throughout (Table A5), which suggests that excluding respondents with incomplete covariate data did not materially bias the association in either direction. That reassurance is conditional, however: multiple imputation assumes data are missing at random given the variables in the imputation model, an assumption that cannot be verified and that multiple imputation cannot rescue if violated [29]. Generalizability to the most socioeconomically vulnerable adults, who were disproportionately excluded, remains limited. A related measurement caveat applies within the analytic sample: 14 respondents (0.26%) have a health literacy score to which at least one unanswered item contributed, so their scores understate measured literacy; three answered no items and sit at the floor of the scale. Thirteen of the fourteen fall in the lowest category, and their mean KHEI was 62.2, above the unweighted sample mean of 60.2; restricting the analysis to the 5339 respondents whose scores reflect answered items left the estimate essentially unchanged (Section 3.6).

Further limitations concern specification and measurement. BMI was intentionally not adjusted to avoid over-adjustment for a variable we judged downstream of diet (Figure A1); residual confounding by unmeasured factors cannot be excluded. Alcohol use was captured with a lifetime use indicator, which may not adequately reflect current drinking patterns relevant to diet quality. Health literacy was measured with a discrete score containing a large modal value, so the score-based categories are necessarily unequal in size and are defined by this sample’s distribution rather than by external criteria; estimates were nevertheless directionally consistent across alternative tie-respecting definitions, the continuous score, and the officially defined binary indicator. Finally, smoking, physical activity, and chronic disease may lie partly downstream of health literacy rather than act solely as confounders, so Model 3 should be interpreted as an extensively adjusted association rather than as an estimate of the total effect of health literacy.

## 5. Conclusions

Among Korean adults, lower health literacy was associated with modestly poorer overall diet quality, with the crude association masked—and reversed in sign—by age until it was adjusted for. The novelty of this work lies not in demonstrating that health literacy and diet are related, which the prior literature has established, but in quantifying that relationship in a nationally representative Korean population with the official Korean Healthy Eating Index, in showing how completely the estimate depends on adjustment for age, and in mapping it across the components of the index and across sex, age, and income. Two findings qualify the result: the association was small (−0.13 SD), and component-level differences, although largest for fruit and vegetables, did not survive correction for multiple testing and remain exploratory, as does the more pronounced association observed in men. Because health literacy is modifiable, it remains a plausible upstream target for population dietary strategies; whether improving it measurably raises diet quality is a question that only longitudinal and interventional research can answer, and one that the present cross-sectional analysis cannot settle.

## Figures and Tables

**Table 1:** Participant characteristics by health literacy category, 2024 KNHANES (n = 5353). Continuous variables are survey-weighted means (survey-weighted SD); categorical variables are unweighted counts with survey-weighted percentages.

Characteristic	≤28 (Lowest)	29–30	31–32	≥33 (Highest)	*p*
Participants, n (%)	1465 (25.5)	2018 (37.0)	480 (9.3)	1390 (28.1)	—
Age, years, mean (SD)	55.0 (17.6)	51.1 (16.3)	45.3 (15.8)	43.5 (15.0)	<0.001
Women, n (%)	841 (48.7)	1101 (48.0)	272 (50.5)	850 (53.2)	0.024
College education or higher, n (%)	317 (28.6)	794 (44.8)	245 (56.5)	822 (62.3)	<0.001
Highest household income quintile, n (%)	231 (16.1)	421 (21.3)	100 (21.6)	343 (23.7)	<0.001
Current smoker, n (%)	228 (19.2)	286 (16.3)	57 (13.7)	148 (12.5)	<0.001
Lifetime alcohol use, n (%)	1232 (88.0)	1844 (93.3)	447 (94.8)	1297 (95.0)	<0.001
Aerobic physical activity, n (%)	525 (40.0)	854 (45.2)	234 (50.6)	752 (56.7)	<0.001
Chronic disease (hypertension or diabetes), n (%)	677 (37.2)	710 (30.6)	117 (20.0)	293 (18.3)	<0.001
Single-person household, n (%)	343 (21.1)	323 (15.3)	81 (17.1)	195 (14.5)	<0.001
Depressive symptoms (PHQ-9 ≥ 10), n (%)	82 (5.8)	76 (4.4)	11 (2.5)	59 (4.0)	0.044
Total energy intake, kcal/day, mean (SD)	1830.8 (858.8)	1902.0 (835.6)	1881.2 (793.0)	1892.9 (814.4)	0.167
KHEI total, mean (SD)	59.2 (13.4)	59.3 (13.1)	57.4 (12.6)	57.8 (12.8)	0.001

Values are survey-weighted means with survey-weighted standard deviations for continuous variables, and unweighted numbers of participants with survey-weighted percentages for categorical variables; all means, standard deviations, and percentages were calculated using the KNHANES nutrition survey weights, strata, and primary sampling unit codes. *p* values were calculated using survey-weighted linear regression for continuous variables and Rao–Scott chi-square tests for categorical variables. Categories are defined by the health literacy score, with all participants sharing a score assigned to the same category; ≤28 points is the lowest and ≥33 points is the highest. Depressive symptoms were assessed in the 5208 participants with a complete PHQ-9. KHEI, Korean Healthy Eating Index; PHQ-9, Patient Health Questionnaire-9; SD, standard deviation.

**Table 2:** Association between health literacy and diet quality (KHEI total), survey-weighted linear and logistic regression, 2024 KNHANES (n = 5353).

Health Literacy	Model 1 (Crude), β (95% CI)	Model 1a (Age Only), β (95% CI)	Model 2, β (95% CI)	Model 3, β (95% CI)
≥33 (highest)	0 (reference)	0 (reference)	0 (reference)	0 (reference)
31–32	−0.39 (−1.78 to 1.01)	−1.05 (−2.28 to 0.19)	−0.91 (−2.13 to 0.30)	−0.75 (−1.96 to 0.46)
29–30	+1.48 (0.49 to 2.47)	−1.29 (−2.18 to −0.39)	−1.04 (−1.94 to −0.15)	−0.82 (−1.69 to 0.04)
≤28 (lowest)	+1.41 (0.37 to 2.44)	−2.76 (−3.72 to −1.79)	−2.10 (−3.07 to −1.12)	−1.70 (−2.66 to −0.75)
Per 1-point increase	—	—	—	+0.15 (0.08 to 0.22)
Per 1-SD increase	—	—	—	+0.77 (0.42 to 1.12)
Official binary indicator, limited (<30) vs. adequate (≥30 points)	—	—	—	−1.06 (−1.80 to −0.32)
Lowest tertile diet quality, lowest vs. highest category	—	—	—	OR = 1.37 (1.09 to 1.72); *p* = 0.007
Lowest tertile diet quality, limited vs. adequate (official binary)	—	—	—	OR = 1.20 (1.01 to 1.42); *p* = 0.041

The last two rows report odds ratios from survey-weighted logistic regression; every other row reports a β coefficient from survey-weighted linear regression. Model 1 was unadjusted. Model 1a was adjusted for age only. Model 2 was adjusted for sex, age, education, household income, marital status, and employment. Model 3 was additionally adjusted for smoking, alcohol use, aerobic physical activity, chronic disease, and single-person household. The four health literacy categories are defined by the observed score distribution and are not clinically validated strata; the per-point, per-SD, and official binary specifications do not depend on those boundaries. The official binary indicator classifies a score below 30 of 40 as limited health literacy (*p* = 0.005 for the KHEI difference and *p* = 0.041 for the odds ratio). Lowest tertile diet quality denotes a KHEI total score in the lowest survey-weighted tertile (≤52.9) of this study population. CI, confidence interval; KHEI, Korean Healthy Eating Index; OR, odds ratio; SD, standard deviation.

## Data Availability

The KNHANES data are publicly available from the Korea Disease Control and Prevention Agency (https://knhanes.kdca.go.kr) (accessed on 20 May 2026).

## Abbreviations

KNHANES	Korea National Health and Nutrition Examination Survey
KHEI	Korean Healthy Eating Index
KDCA	Korea Disease Control and Prevention Agency
PHQ-9	Patient Health Questionnaire-9
DAG	directed acyclic graph
FDR	false discovery rate
BMI	body mass index
CI	confidence interval
OR	odds ratio
SD	standard deviation

## Appendix A

### Appendix A.1

**Table A1:** Associations between health literacy and individual components of the Korean Healthy Eating Index (KHEI), fully adjusted model. Because the maximum attainable score differs across components, differences are also shown as a percentage of each component’s maximum score.

Domain	KHEI Component	β (95% CI)	% of Maximum (Maximum Score)	*p* Value	FDR-Adjusted *p* Value
Adequacy	Having breakfast	−0.25 (−0.60 to 0.10)	−2.5 (10)	0.165	0.289
	Mixed grains	−0.07 (−0.25 to 0.12)	−1.4 (5)	0.476	0.667
	Total fruit	−0.18 (−0.35 to −0.01)	−3.7 (5)	0.035	0.161
	Fresh fruit	−0.23 (−0.41 to −0.05)	−4.6 (5)	0.014	0.161
	Total vegetables	−0.02 (−0.15 to 0.11)	−0.5 (5)	0.714	0.714
	Vegetables (excluding kimchi/pickled vegetables)	−0.17 (−0.31 to −0.02)	−3.3 (5)	0.026	0.161
	Meat, fish, eggs, and beans	−0.20 (−0.44 to 0.05)	−2.0 (10)	0.115	0.289
	Milk and dairy	−0.23 (−0.63 to 0.17)	−2.3 (10)	0.255	0.397
Moderation	Saturated fat (% energy)	+0.08 (−0.33 to 0.48)	+0.8 (10)	0.710	0.714
	Sodium	−0.08 (−0.36 to 0.19)	−0.8 (10)	0.549	0.699
	Sweets and beverages (% energy)	+0.07 (−0.27 to 0.41)	+0.7 (10)	0.666	0.714
Balance	Carbohydrate (% energy)	−0.13 (−0.32 to 0.05)	−2.6 (5)	0.164	0.289
	Fat (% energy)	−0.14 (−0.33 to 0.06)	−2.8 (5)	0.161	0.289
	Energy intake	−0.15 (−0.36 to 0.06)	−3.0 (5)	0.152	0.289

CI, confidence interval; FDR, false discovery rate; KHEI, Korean Healthy Eating Index. Estimates represent adjusted mean differences in KHEI component scores for the lowest versus highest health literacy category (≤28 vs. ≥33 points) from the fully adjusted survey-weighted linear regression model. Models were adjusted for age, sex, education, household income, marital status, employment, smoking, alcohol use, aerobic physical activity, chronic disease, and single-person household. *p* values were corrected for multiple testing using the Benjamini–Hochberg false discovery rate procedure.

### Appendix A.2

**Table A2:** Subgroup analyses of the association between health literacy and diet quality (KHEI total score).

Stratification	Level	β (95% CI)	*p* Value	Interaction *p* Value
Age	19–39 years	−1.15 (−3.21 to 0.91)	0.272	0.354
	40–64 years	−2.25 (−3.69 to −0.81)	0.002	
	≥65 years	−1.09 (−3.05 to 0.87)	0.273	
Sex	Men	−2.74 (−4.13 to −1.35)	<0.001	0.025
	Women	−0.75 (−2.01 to 0.51)	0.242	
Household income	Lowest	−2.46 (−4.43 to −0.49)	0.015	0.431
	Lower middle	−1.69 (−3.81 to 0.43)	0.118	
	Middle	−0.68 (−3.02 to 1.66)	0.568	
	Upper middle	−2.22 (−5.00 to 0.56)	0.116	
	Highest	−0.91 (−3.33 to 1.52)	0.462	

CI, confidence interval; KHEI, Korean Healthy Eating Index. Estimates represent adjusted mean differences in KHEI total score comparing the lowest with the highest health literacy category (≤28 vs. ≥33 points). Models were adjusted for age, sex, education, household income, marital status, employment, smoking, alcohol use, aerobic physical activity, chronic disease, and single-person household, except for the stratification variable. Because the four-category exposure contributes three interaction terms, the interaction column reports the joint Wald *p* value for those terms rather than a single coefficient; the interaction estimate with its 95% confidence interval is given for the continuous specification in Section 3.5.

### Appendix A.3

**Table A3:** Comparison of respondents included in and excluded from the primary complete case analysis, 2024 KNHANES.

Group	Respondents, n	Score Not Reflecting Answered Items, n	Health Literacy Score, Mean (SD)	Age, Years, Mean (SD)	Crude KHEI, Mean (SD)
Included in the primary analysis	5353	14	30.4 (4.9)	53.5 (17.1)	60.2 (13.1)
Excluded: no nutrition survey weight	174	68	29.4 (5.4)	55.3 (14.1)	Not computable
Excluded: KHEI not computable	3	2	22.0	68.0	Not computable
Excluded: missing ≥1 covariate	503	164	29.1 (5.5)	56.6 (16.5)	60.7 (12.0)

Values are unweighted means (SD), because respondents excluded for lacking a nutrition survey weight cannot be placed on the same weighted scale as the analytic sample. Crude KHEI is shown only for the groups in which it could be computed, and dispersion is not shown where fewer than two respondents contribute. The summary health literacy score distributed with the survey enters a “don’t know or no response” item code as a value of one, so respondents who did not answer the items receive the minimum score of 10; the third column counts those respondents together with the two for whom no score was recorded (246 and 2, respectively, across all groups), and the health literacy, age, and KHEI columns exclude them so that the comparison reflects measured health literacy. KHEI, Korean Healthy Eating Index; SD, standard deviation.

### Appendix A.4

**Table A4:** Exclusion from the complete case analysis by health literacy category, 2024 KNHANES.

Health Literacy Category	Respondents with a Measured Score, n	Excluded, n	Exclusion Rate, %	Share of All Exclusions, %
≤28 points (lowest)	1609	157	9.8	35.2
29–30 points	2188	171	7.8	38.3
31–32 points	505	25	5.0	5.6
≥33 points (highest)	1483	93	6.3	20.9
Total	5785	446	7.7	100.0

Denominators are the 5785 respondents aged ≥19 years whose health literacy score reflects answered items; the 248 respondents without a measured score are excluded, as explained in the footnote to Table A3 and in Section 2.2. Exclusion rate is the percentage of respondents in that category who were excluded from the complete case analysis for any reason; share of all exclusions is the percentage of the 446 excluded respondents with a measured score who fell in that category. Category sizes, therefore, differ slightly from those in Table 1, which describes the analytic sample using the score as supplied.

### Appendix A.5

**Table A5:** Multiple imputation sensitivity analysis compared with the primary complete case analysis.

Specification	Complete Case (n = 5353)	Multiple Imputation (n = 5678, m = 20)
KHEI, ≤28 vs. ≥33 points, β (95% CI)	−1.70 (−2.66 to −0.75)	−1.66 (−2.57 to −0.74)
KHEI, 29–30 vs. ≥33 points, β (95% CI)	−0.82 (−1.69 to 0.04)	−0.97 (−1.80 to −0.13)
KHEI, 31–32 vs. ≥33 points, β (95% CI)	−0.75 (−1.96 to 0.46)	−0.76 (−1.92 to 0.41)
KHEI, per 1-SD increase, β (95% CI)	+0.77 (0.42 to 1.12)	+0.76 (0.44 to 1.08)
KHEI, official binary (<30 vs. ≥30), β (95% CI)	−1.06 (−1.80 to −0.32)	−0.97 (−1.67 to −0.27)
Lowest tertile diet quality, ≤28 vs. ≥33 points, OR (95% CI)	1.37 (1.09 to 1.72)	1.33 (1.07 to 1.64)
Lowest tertile diet quality, 29–30 vs. ≥33 points, OR (95% CI)	1.22 (1.01 to 1.46)	1.22 (1.03 to 1.45)
Lowest tertile diet quality, 31–32 vs. ≥33 points, OR (95% CI)	1.17 (0.88 to 1.55)	1.15 (0.88 to 1.51)
Lowest tertile diet quality, official binary, OR (95% CI)	1.20 (1.01 to 1.42)	1.15 (0.98 to 1.35)

Complete case estimates are from the analytic sample of 5353 respondents. Multiple imputation estimates pool 20 imputed datasets generated among the 5678 respondents who had a nutrition survey weight, a computable KHEI, and a health literacy score reflecting answered items, combined using Rubin’s rules; respondents whose score was assembled from unanswered items are excluded from the imputation set for the reason given in Section 2.2. A further analysis that also imputed the health literacy score, across all 5856 respondents with a nutrition survey weight and a computable KHEI, gave closely similar results (lowest versus highest category, −1.67; 95% CI, −2.58 to −0.75; per 1-SD, +0.77; 95% CI, 0.45 to 1.09). All models are fully adjusted (Model 3) and account for the KNHANES complex survey design. Lowest tertile diet quality denotes a KHEI total score in the lowest survey-weighted tertile (≤52.9) of this study population. CI, confidence interval; KHEI, Korean Healthy Eating Index; OR, odds ratio; SD, standard deviation.
